# *Lilium regale* Wilson WRKY2 Regulates Chitinase Gene Expression During the Response to the Root Rot Pathogen *Fusarium oxysporum*

**DOI:** 10.3389/fpls.2021.741463

**Published:** 2021-09-27

**Authors:** Shan Li, Jun Hai, Zie Wang, Jie Deng, Tingting Liang, Linlin Su, Diqiu Liu

**Affiliations:** Faculty of Life Science and Technology, Kunming University of Science and Technology, Kunming, China

**Keywords:** *Lilium regale*, *Fusarium oxysporum*, WRKY transcription factors, transcriptional regulation, W-box, chitinase

## Abstract

Root rot, mainly caused by *Fusarium oxysporum*, is the most destructive disease affecting lily (*Lilium* spp.) production. The WRKY transcription factors (TFs) have important roles during plant immune responses. To clarify the effects of WRKY TFs on plant defense responses to pathogens, a WRKY gene (*LrWRKY2*) was isolated from *Lilium regale* Wilson, which is a wild lily species highly resistant to *F. oxysporum*. The expression of *LrWRKY2*, which encodes a nuclear protein, is induced by various hormones (methyl jasmonate, ethephon, salicylic acid, and hydrogen peroxide) and by *F. oxysporum* infection. In this study, *LrWRKY2*-overexpressing transgenic tobacco plants were more resistant to *F. oxysporum* than the wild-type plants. Moreover, the expression levels of jasmonic acid biosynthetic pathway-related genes (*NtAOC, NtAOS, NtKAT, NtPACX, NtJMT, NtOPR*, and *NtLOX*), pathogenesis-related genes (*NtCHI, NtGlu2*, and *NtPR-1*), and antioxidant stress-related superoxide dismutase genes (*NtSOD, NtCu-ZnSOD*, and *MnSOD*) were significantly up-regulated in *LrWRKY2* transgenic tobacco lines. Additionally, the transient expression of a hairpin RNA targeting *LrWRKY2* increased the susceptibility of *L. regale* scales to *F. oxysporum*. Furthermore, an *F. oxysporum* resistance gene (*LrCHI2*) encoding a chitinase was isolated from *L. regale*. An electrophoretic mobility shift assay demonstrated that LrWRKY2 can bind to the *LrCHI2* promoter containing the W-box element. Yeast one-hybrid assay results suggested that LrWRKY2 can activate *LrCHI2* transcription. An examination of transgenic tobacco transformed with *LrWRKY2* and the *LrCHI2* promoter revealed that LrWRKY2 activates the *LrCHI2* promoter. Therefore, in *L. regale*, LrWRKY2 is an important positive regulator that contributes to plant defense responses to *F. oxysporum* by modulating *LrCHI2* expression.

## Introduction

Plants have evolved a series of responsive and adaptive mechanisms to cope with various stresses. The WRKY transcription factors (TFs), which represent one of the largest families of transcriptional regulators in plants, are mainly involved in regulating the plant immune system during responses to stress (Wang P. et al., 2018). These TFs usually contain one or two conserved domains comprising approximately 60 amino acids (Yan et al., 2019a). The domain with a highly conserved WRKYGQK motif at the N-terminal end and a zinc-finger motif (CX_4−5_CX_22−23_HXH or CX_7_CX_23_HXC) at the C-terminal end was designated as the WRKY domain (Jimmy and Babu, 2019). On the basis of the number of WRKY domains and the structure of the zinc-finger motif, WRKY TFs have been classified into three main groups (I, II, and III) (Dong et al., 2020). The Group I members have two WRKY domains and a C_2_H_2_ (CX_4−5_CX_22−23_HXH) zinc-finger motif, the Group II members contain one WRKY domain and a C_2_H_2_ (CX_4−5_CX_22−23_HXH) zinc-finger motif, and the Group III members contain a single WRKY domain and a C_2_HC (CX_7_CX_23_HXC) motif (Nan and Gao, 2019). Moreover, the Group II WRKY TFs have been further divided into Subgroups IIa, IIb, IIc, IId, and IIe according to their primary amino acid sequences (Huang et al., 2015).

Transcription factors bind to *cis*-acting elements in the promoters of their downstream target genes to regulate expression (Long et al., 2019). The WRKY TFs are a family of plant-specific DNA-binding proteins (Rushton et al., 2010). More specifically, the WRKY domain binds to the W-box (TTGACC/T) in the promoters of target genes to activate, repress, or de-repress transcription. WRKY TFs can also bind to several W-box-like elements (Choi et al., 2015). It is due to WRKY TFs binds to the TGAC core motif of the W-box, whereas the preferred WRKY TF-binding sites are determined by the sequences adjacent to the TGAC core motif (Choi et al., 2015). The WRKYGQK and zinc-finger motifs are required for the high-affinity binding of WRKY proteins to the consensus *cis*-element W-box present in the promoters of target genes, including those encoding pathogenesis-related (PR) proteins (Karim et al., 2015). The solution structure of the WRKY domain was first reported by Yamasaki et al. (2005). The WRKY domain of the *Arabidopsis thaliana* WRKY4 TF comprises a four-stranded β-sheet, with zinc-coordinating Cys/His residues forming a zinc-binding pocket (Yamasaki et al., 2005). The WRKYGQK residues form the most N-terminal β-strand, which partly protrudes from the surface of the protein, enabling it to interact with the DNA major groove. The β-strand including the WRKYGQK motif reportedly interacts with an approximately 6-bp region, which is consistent with the length of the W-box (Jiang et al., 2016). The WRKYGQK motif recognizes DNA mainly through apolar associations with the methyl groups of the thymine bases present in the W-box which was reviewed by Viana et al. (2018).

The WRKY TFs have a very complex regulatory role influencing plant disease resistance. They are central components mediating innate immunity, including molecular pattern-triggered immunity, pathogen-associated molecular pattern-triggered immunity, effector-triggered immunity, basal defense, and systemic acquired resistance (Eulgem and Somssich, 2007). The activation of WRKY TFs under stress conditions leads to a thorough transcriptional reprogramming affecting defense-related genes, including genes for secondary metabolites (phytoalexins such as momilactones and phenylamides) and PR proteins (PR3, PR4c, PR10a, PR10b, and root-specific PR10) (Jimmy and Babu, 2019). The constitutive expression of rice (*Oryza sativa*) *WRKY45* in *A. thaliana* enhances the resistance of the transgenic plants to the pathogen *Pseudomonas syringae* pv. *tomato* DC3000 by up-regulating the expression of some *PR* genes (Qiu and Yu, 2009). In *Capsicum annuum*, WRKY40b is a negative regulator that directly affects immunity at multiple levels by modulating the production of signal regulatory proteins, TFs, and PR proteins (Ifnan Khan et al., 2018). In a previous study, the overexpression of apple (*Malus* × *domestica*) *WRKYN1* in the susceptible apple cultivar “Golden Delicious” resulted in increased resistance to the apple leaf spot fungus *Alternaria alternata* f. sp. *mali*, which was related to the significantly up-regulated expression of *PR* genes (Zhang et al., 2017).

Root rot caused by *Fusarium oxysporum* seriously threatens the growth and development of lily (*Lilium* spp.). Several wild *Lilium* species are highly resistant to *F. oxysporum*, including *L. regale* Wilson, *L. pumilum*, and *L. dauricum* Ker Gawler (He et al., 2014, 2019; Ramekar et al., 2019). Although the germplasm resources of *Lilium* species resistant to *F. oxysporum* have been used for breeding disease-resistant lines, little is known about their mechanisms regulating transcription during an infection by *F. oxysporum*. In a recent study, 35 WRKY genes were identified in *L. regale* after the transcriptomes of plants inoculated with *F. oxysporum* were sequenced (Li et al., 2021b). At the transcriptional level, *LrWRKY2* is highly responsive to methyl jasmonate (MeJA), salicylic acid (SA), ethephon (ETH), and hydrogen peroxide (H_2_O_2_) treatments as well as *F. oxysporum* infections. Thus, in this study, we focused on unraveling the *LrWRKY2* (accession no. MW125547) regulatory mechanism during the *L. regale* defense response to *F. oxysporum*. The tobacco is an important cash crop and model plant used for rapid verification of plant gene functions, moreover, the *F. oxysporum* causes root rot in tobacco (Clemente, 2006; Ding et al., 2021). In addition, the infection rate of *F. oxysporum* in tobacco seedlings is faster than the lily, and the obvious disease symptoms can appear within a week after inoculation. Therefore, the effects of LrWRKY2 on the resistance to *F. oxysporum* was evaluated by overexpressing *LrWKKY2* in tobacco plants and transiently expressing the hairpin RNA targeting *LrWRKY2* in *L. regale* scales considering the long period of lily genetic transformation and great difficulties to obtain enough transformants for subsequent experiments. Furthermore, LrWRKY2 was characterized regarding its subcellular localization and its ability to activate the promoter of an *L. regale PR* gene (*LrCHI2*), which is an *F. oxysporum-*responsive gene according to transcriptome sequencing data. Additionally, tobacco plants were transformed with the *LrCHI2* promoter and *LrWRKY2* to analyze the regulatory effects of LrWRKY2 on *LrCHI2* expression.

## Materials and Methods

### Plant and Fungal Materials

Wild *L. regale* Wilson plants collected in the Minjiang River Basin of Sichuan province, China, were grown in a greenhouse. An *F. oxysporum* strain isolated from diseased lily plants exhibiting typical symptoms of Fusarium wilt was characterized and preserved by our research group. Several typical plant fungal pathogens (*Colletotrichum gloeosporioides, Fusarium solani*, and *Alternaria panax*) were also selected for the antifungal activity analysis. *F.oxysporum, C. gloeosporioides, F. solani*, and *A. panax* stored at 4°C were cultured on potato dextrose agar (PDA) before use. Sterile tobacco seedlings were cultured in a climate-controlled cabinet prior to genetic transformations.

### Gene Cloning

The total RNA was extracted from 1 g wild *L. regale* roots using the TRIGene kit (Genstar, China). The cDNA was obtained following the reverse transcription of total RNA using the GoScript™ Reverse Transcription System (Promega, USA) and served as the template for isolating *LrWRKY2* and *LrCHI2*. Gene-specific primers (Supplementary Table 1) were designed to amplify the *LrWRKY2* and *LrCHI2* open reading frames (ORFs) using *L. regale* cDNA as the template. The PCR products were cloned into the pMD-18T vector (TaKaRa, Japan) and the resulting recombinant vectors pMD-18T-*LrWRKY2* and pMD-18T-*LrCHI2* were inserted into competent *Escherichia coli* DH5α cells. The positive clones on a LB plate adding the ampicillin (50 mg/L) were verified by PCR and sequencing in Tsingke Biotechnology Co., Ltd. Bioinformatics analyses were completed as described by Taif et al. (2020).

### Quantitative Real-Time PCR (qRT-PCR)

The *LrCHI2* expression pattern in *L. regale* was analyzed in a qRT-PCR assay. The roots, leaves, stems, flowers, and scales of healthy *L. regale* plants were used to analyze tissue-specific *LrCHI2* expression. Regarding the fungal inoculation, the root tips were wounded using surgical scissors and inoculated with a fresh *F. oxysporum* spore suspension (5 × 10^6^ spores/mL) for 30 min. The control plants were treated with sterile water instead of the spore suspension. The roots were sampled at 12, 24, 48, and 72 h post-inoculation. Finally, total RNA extracted from each sample was reverse transcribed into cDNA for a qRT-PCR analysis of *LrCHI2* expression levels using the GoScript™ Reverse Transcription System (Promega, USA). The SYBR green I-based qRT-PCR analysis was conducted using the ABI Prism 7500 Sequence Detection System (Applied Biosystems, USA) and Eastep® qPCR Master Mix (Promega, USA) according to an established method (Zhao et al., 2020). The *L. regale* glyceraldehyde-3-phosphate dehydrogenase gene (*LrGAPDH*, GenBank No. KJ543468.1) was used as an internal reference gene to calculate the relative expression values of *LrCHI2*. The *LrCHI2* expression levels were calculated according to the 2^−ΔΔCt^ method. The qRT-PCR analysis was completed using three biological replicates, and there were three technical repetitions included in each biological replicate.

### Subcellular Localization

The subcellular localization of LrWRKY2 and LrCHI2 was predicted using the PSORT online program (https://www.genscript.com/psort.html) and then confirmed by transiently expressing green fluorescent protein (GFP)-tagged fusion proteins in onion (*Allium cepa*) epidermal cells. More specifically, the *LrWRKY2* and *LrCHI2* ORFs lacking the stop codon were amplified from pMD-18T-*LrWRKY2* and pMD-18T-*LrCHI2*, respectively. The PCR products were then inserted into the pBIN m0-gfp5-ER vector to generate the *LrWRKY2-GFP* and *LrCHI2-GFP* constructs. The two recombinant vectors were transferred into *Agrobacterium tumefaciens* EHA105 cells using a CaCl_2_ freeze–thaw method (Holsters et al., 1978). An empty pBIN m-gfp5-ER vector served as a control. The transformed *A. tumefaciens* cells were used to insert the recombinant or control vector into onion epidermal cells, which were then cultured in darkness for 48 h. To analyze transient gene expression, GFP fluorescence in the onion epidermal cells was monitored using a confocal microscope (Nikon, JPN) as described by Liu et al. (2018).

### Generation and Screening of Transgenic Tobacco Lines Overexpressing *LrWRKY2* or *LrCHI2*

The plasmids pMD-18T-*LrWRKY2* and pCAMBIA2300s were digested with *Bam*HI and *Xba*I, while the pMD-18T-*LrCHI2* and pCAMBIA2300s plasmids were digested with *Bam*HI and *Eco*RI for constructing the overexpression vectors of *LrWRKY2* and *LrCHI2*, respectively. After which the pCAMBIA2300s-*LrWRKY2* and pCAMBIA2300s-*LrCHI2* recombinant plasmids were generated using the T4 DNA ligase. The recombinant plasmids were inserted into separate competent *A. tumefaciens* LBA4404 cells. The positive clones identified by PCR were used to transform tobacco leaf disks as described by Horsch et al. (1985). Genomic DNA was extracted from T_0_ transgenic tobacco plants using cetyltrimethylammonium bromide (CTAB) as described by Allen et al. (2006), and then the genomic DNA was used as the template for a PCR conducted to confirm the transgenic lines carried the correct transgene, with wild-type (WT) plants serving as the negative control. The transgenic tobacco plants were grown in a greenhouse to produce T_2_ generation lines.

### Analysis of Gene Expression Levels and Evaluation of the Disease Resistance of T_2_ Transgenic Tobacco Plants

Total RNA was extracted from T_2_ generation *LrWRKY2* transgenic tobacco lines and reverse transcribed into cDNA with the forementioned method. The *LrWRKY2* transcription levels in the transgenic tobacco lines were determined by qRT-PCR with the tobacco actin gene (*NtACT*, GenBank No. AB158612.1) as an internal reference gene. Moreover, the roots and leaves of several *LrWRKY2* transgenic tobacco lines and WT plants were inoculated with *F. oxysporum* to evaluate the disease resistance of the transgenic tobacco lines. After the wounded tobacco roots were immersed in an *F. oxysporum* spore suspension (5 × 10^6^ spores/mL) for 30 min, the inoculated tobacco plants were grown under hydroponic conditions in an illumination incubator for 1 week. Regarding the leaf inoculation, leaves were wounded, inoculated with 20 μL *F. oxysporum* spore suspension (5 × 10^6^ spores/mL), and then placed in a humid illumination incubator for 1 week. The leaves of T_2_ generation *LrCHI2* transgenic tobacco lines were similarly inoculated. The disease symptoms caused by the *F. oxysporum* infections were examined.

The expression levels of the following defense-related genes in the *LrWRKY2* transgenic tobacco lines were analyzed by qRT-PCR with the forementioned method: jasmonic acid (JA) biosynthetic pathway-related genes (*NtAOC, NtAOS, NtKAT, NtPACX, NtJMT, NtOPR*, and *NtLOX*), *PR* genes (*NtCHI, NtGlu2*, and *NtPR-1*), and antioxidant stress-related superoxide dismutase (SOD) genes (*NtSOD, NtCu-ZnSOD*, and *MnSOD*). Sequence details regarding the defense-related genes were obtained from the NCBI database (https://www.ncbi.nlm.nih.gov/) and used to design gene-specific primers (Supplementary Table 2). Three *LrWRKY2* transgenic tobacco lines were randomly selected for an analysis of the expression of these defense-related genes.

### Transient Expression of Hairpin RNA Targeting *LrWRKY2* in *L. regale*

Primers with the attB linker were designed to amplify the *LrWRKY2* RNA interference (RNAi) fragment (Supplementary Table 1). The PCR product was incorporated into the RNAi vector pHellsgate2 via a BP recombination reaction using the Gateway® BP Clonase™ II Enzyme Mix kit (Invitrogen, USA). The pHellsgate2-*LrWRKY2* recombinant plasmid was inserted into competent *E. coli* DH10B cells. Positive clones were selected on agar-solidified Luria-Bertani medium containing spectinomycin (90 mg/L). The plasmids of the positive clones were digested with *Xba*I and *Xho*I to confirm the *LrWRKY2* fragment was correctly recombined. The pHellsgate2-*LrWRKY2* recombinant plasmid and the empty pHellsgate2 vector were inserted into separate *A. tumefaciens* EHA105 cells, which were then cultured in MGL medium at 28°C for 5 h in a constant-temperature shaker (150 rpm). Fresh *L. regale* scales were washed with sterile water and rubbed with sandpaper to form uniformly sized wounds. The wounded *L. regale* scales were transformed with *A. tumefaciens* liquid containing the pHellsgate2-*LrWRKY2* recombinant plasmid or the empty pHellsgate2 vector. The *L. regale* scales were placed on filter paper moistened with sterile water and incubated in a climate-controlled cabinet set at 25°C for 24 h, and then inoculated with 20 μL *F. oxysporum* spore suspension (5 × 10^6^ spores/mL). The inoculated scales were collected at 72 h for an analysis of *LrWRKY2* expression and disease symptoms. The *L. regale* scales in which the empty pHellsgate2 vector was transiently expressed were used as the control.

### Expression and Purification of the Recombinant LrWRKY2 and LrCHI2 Proteins

The SignalP 5.0 Server (https://www.cbs.dtu.dk/services/SignalP/) was used for predicting the presence of a signal peptide. The *LrWRKY2* ORF amplified by PCR from pMD-18T-*LrWRKY2* was digested with *Hin*dIII and *Bam*HI and then subcloned into the pET-32a vector. Gene-specific primers (Supplementary Table 1) were designed to amplify the *LrCHI2* ORF without the signal peptide-encoding sequence. The PCR product was inserted into pMD-18T. The resulting recombinant plasmid was digested with *Eco*RI and *Eco*RV and then the *LrCHI2* fragment was incorporated into the pET-32a vector. The pET-32a-*LrWRKY2* and pET-32a-*LrCHI2* plasmids were transferred into competent *E. coli* BL21 cells. The empty pET-32a vector was used as a control. The production of the LrWRKY2 and LrCHI2 recombinant proteins was induced and the inclusion body proteins were denatured and renatured as described by Zhao et al. (2020). The recombinant proteins were purified using the Ni-NTA Sepharose Column (Sangon Biotech, China).

### Analysis of the Antifungal Activity of the Recombinant LrCHI2 Protein

The *in vitro* antifungal effects of the LrCHI2 recombinant protein were examined using *F. oxysporum, C. gloeosporioides, F. solani*, and *A. panax*. Agar plugs (1 cm diameter) removed from the outer edge of actively growing fungus on agar-solidified PDA medium in plates were used to inoculate fresh agar-solidified PDA medium. When the diameter of the fungal colonies reached 2 cm, LrCHI2 (5, 10, and 20 μg) was added to the plates. Sterile water and phosphate buffer (pH 8.0) were used as controls. After a 3-day incubation at 28°C, the fungal colonies were photographed and the average growth inhibition zones (mm^2^) were calculated using Photoshop 7.0.

### Cloning of the *LrCHI2* Promoter Fragment

The *LrCHI2* promoter region was isolated from *L. regale* genomic DNA by genome walking using the Universal GenomeWalker™ 2.0 kit (TaKaRa, Japan). Two nested primers were designed specifically for the *LrCHI2* unigene sequence (Supplementary Table 1). The sequence upstream of *LrCHI2* was amplified by two rounds of PCR. The resulting PCR product was cloned into pMD-18T. The promoter fragment was confirmed by sequencing and an alignment with the *LrCHI2* unigene sequence, and a 491-bp *LrCHI2* promoter fragment was obtained. Additionally, the promoter *cis*-elements were predicted using the PlantCARE program (http://bioinformatics.psb.ugent.be/webtools/plantcare/html/).

### Electrophoretic Mobility Shift Assay (EMSA)

A 50-bp *LrCHI2* promoter fragment with one W-box was used as the probe. The mutant probe had one mutation in the W-box core sequence (Supplementary Table 1). These two probe sequences were synthesized and labeled with biotin (Sangon Biotech, China). The unlabeled probe served as the competitor. The EMSA reaction mixtures contained 0.5 μg purified LrWRKY2 protein and 7 μL 1 × gel shift binding buffer [100 mM Tris, 500 mM KCl, 10 mM DTT, 50% glycerol, 100 mM MgCl_2_, 1% NP-40, 1 M KCl, 200 mM EDTA, and 1 μg/μL poly (dI•dC)] in a total volume of 20 μL. After a 20-min incubation at room temperature, 2 μL biotin-labeled probes were added and the incubation was continued for 20 min. For the competitor probe, the binding reaction components were incubated for 40 min. The DNA–protein complexes were electrophoresed on 6.5% non-denaturing polyacrylamide gels in an ice-cold water bath and then transferred to a nylon membrane. Finally, the membrane was dried and analyzed using the LightShift Chemiluminescent EMSA Kit (Pierce, USA).

### Yeast One-Hybrid (Y1H) Assay

A Y1H assay was performed to analyze the *LrCHI2* promoter-binding capability of LrWRKY2. The recombinant prey (pGADT7-*LrWRKY2*) and bait (*pLrCHI2*-pAbAi) vectors were constructed. The reporter strains were generated by integrating linearized *pLrCHI2*-pAbAi or empty pAbAi plasmids into the genome of yeast strain Y1HGold. Positive clones identified by PCR were used to inoculate synthetic defined (SD) medium containing different aureobasidin A (AbA) concentrations to determine the appropriate AbA inhibitory concentration. The recombinant prey plasmid (pGADT7-*LrWRKY2*) was integrated into yeast cells containing *pLrCHI2-*pAbAi or empty pAbAi, whereas the recombinant prey plasmid (pGADT7-*p53*) was inserted into Y1HGold yeast cells carrying the *p53*-pAbAi plasmid (positive control). Positive clones were confirmed by PCR. The protein–DNA interaction was identified on the basis of the activation of the AbA resistance gene when a prey protein from the library bound to the bait sequence. Thus, the three types of Y1HGold yeast cells were used to inoculate SD/–Leu medium supplemented with the appropriate AbA inhibitory concentration. The Y1H assay was performed using the Matchmaker™ Gold Yeast One-Hybrid Library Screening System (Takara, USA).

### Transformation of Tobacco With the *LrCHI2* Promoter and *LrWRKY2*

The *LrCHI2* promoter was cloned to study whether LrWRKY2 regulates *LrCHI2* expression. The pMD-18T-*pLrCHI2* plasmid was digested with *Bam*HI and *Hin*dIII and then the *LrCHI2* promoter fragment was subcloned into the pBI121-*GUS* vector digested with the same restriction enzymes. The pBI121-*pLrCHI2-GUS* plasmid was integrated into WT and *LrWRKY2* overexpressing tobacco leaf disks via *A. tumefaciens*-mediated transformation. As controls, WT and *LrWRKY2* transgenic tobacco leaf disks were transformed with the empty pBI121-*GUS* vector. The genomic DNA of the putative transgenic tobacco lines served as the template for a PCR amplification using β-glucuronidase (GUS) gene-specific primers to verify the transgenic plants were transformed correctly (Supplementary Table 1). The GUS enzyme activity (pM 4-MU min^−1^ μg^−1^ protein) of the confirmed transgenic tobacco lines was analyzed using a fluorescence spectrophotometer (Hitachi F-4600, Japan) as described by Chen et al. (2017).

### Data Analysis

Relative gene expression levels, fungal growth inhibition zone sizes, tobacco leaf and lily scale lesion sizes, and GUS enzyme activities are herein presented as the mean ± standard deviation. The data were analyzed using the SPSS software (version 17.0) and the significance of any differences was determined according to Student's *t-*test and Duncan's multiple range test. All experiments were performed independently at least three times under the same conditions.

## Results

### The *L. regale* Subgroup IIe *WRKY2* Gene Encodes a Nuclear Protein

**Figure 1:**
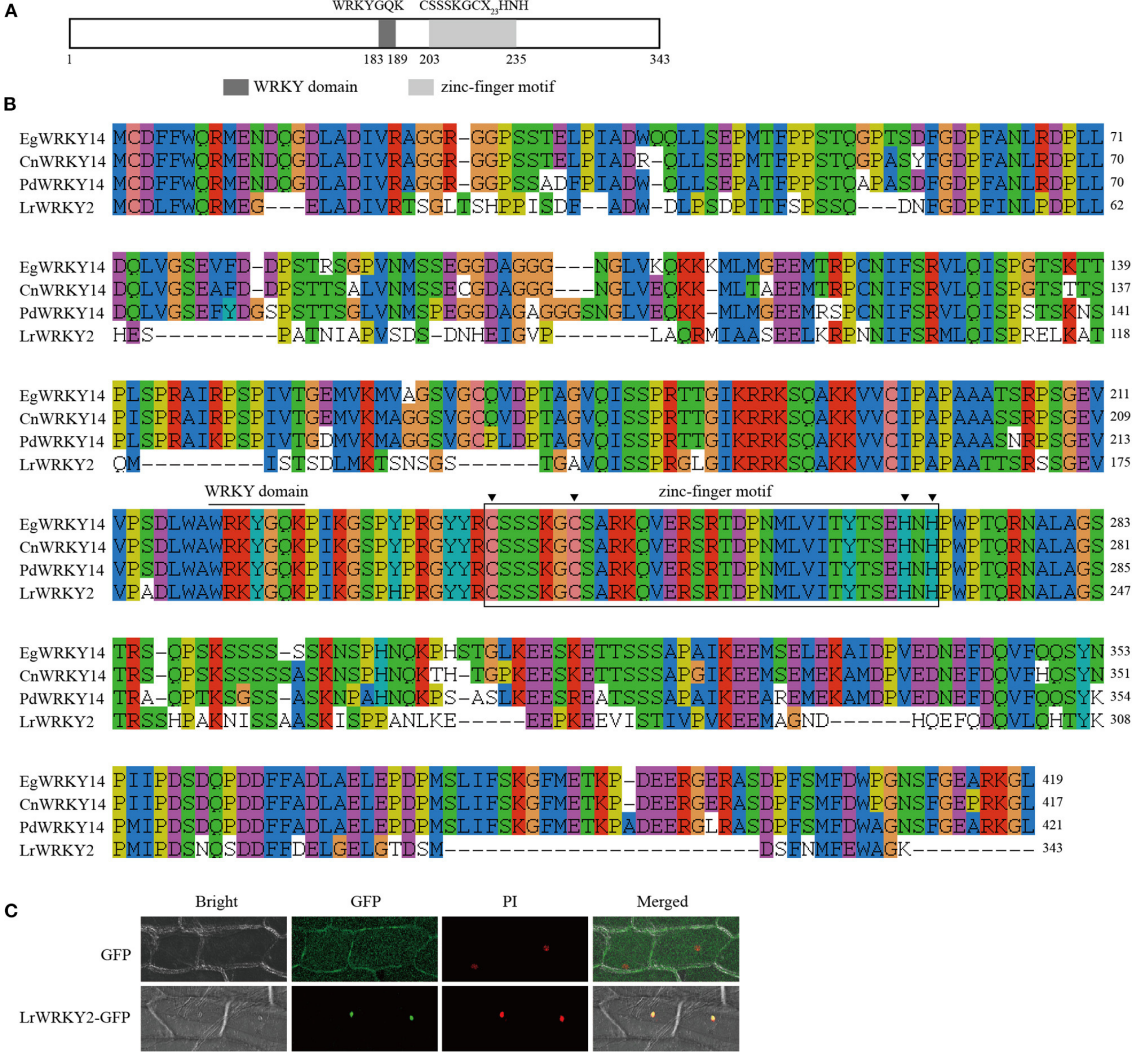
The sequence and subcellular localization analyses of LrWRKY2. **(A)** The conserved domains predicted in the LrWRKY2. **(B)** The multiple alignment of the amino acid sequence of LrWRKY2 and three homologous WRKYs were performed with the ClustalW. **(C)** Subcellular localization analysis of LrWRKY2-GFP fusion protein. The LrWRKY2-GFP fusion protein was transiently expressed in nucleus after genetic transform mediated by *Agrobacterium tumefaciens*. GFP, fluorescent light; Bright, white light; PI, the nucleus was displayed by propidium iodide (PI) staining; Merged, overlaid of fluorescent and the nucleus displayed by PI staining.

Researchers previously identified 35 WRKY genes by sequencing the wild *L. regale* transcriptome during an *F. oxysporum* infection; they also revealed that *LrWRKY2* expression is induced by *F. oxysporum* and is substantially affected by MeJA, SA, ETH, and H_2_O_2_ treatments (Li et al., 2021a). Thus, *LrWRKY2* was functionally characterized. The *L. regale LrWRKY2* cDNA was amplified by RT-PCR using gene-specific primers. The full-length *LrWRKY2* cDNA comprised 1,302 bp, including a 1,032-bp ORF encoding a Subgroup IIe WRKY protein consisting of 343 amino acid residues. A sequence analysis confirmed the putative LrWRKY2 protein contained a typical WRKY domain with the highly conserved WRKYGQK sequence and a C_2_H_2_-type (CX_4−5_CX_22−23_HXH) zinc-finger motif, which is a typical feature of Group II members (Figure 1A). The deduced LrWRKY2 amino acid sequence was highly similar to the sequences of several WRKY proteins from monocots, including *Phoenix dactylifera* WRKY14 (accession no. XP_008788702.2), *Elaeis guineensis* WRKY14 (accession no. XP_010920047.1), and *Cocos nucifera* WRKY14 (accession no. KAG1354178.1) (Figure 1B).

The PSORT program predicted that LrWRKY2 contains a putative NLS “KRPR” sequence, implying LrWRKY2 may be localized in the nucleus. To experimentally determine the subcellular localization of LrWRKY2, the *LrWRKY2* coding sequence was fused to a GFP reporter gene, and the resulting fusion construct was inserted into onion epidermal cells. Green fluorescence was detected throughout the onion cells infected with *A. tumefaciens* carrying the empty pBIN m-gfp5-ER vector, whereas it was limited to the nucleus in cells infected with *A. tumefaciens* carrying the *LrWRKY2-GFP* construct. Moreover, the LrWRKY2-GFP fusion protein colocalized with the nuclear localization marker PI (Figure 1C). These observations indicate LrWRKY2 is a nuclear protein.

### The Overexpression of *LrWRKY2* in Tobacco Increases the Resistance to *F. oxysporum* and Activates Some JA Biosynthesis-Related Genes, *PR* Genes, and SOD Genes

**Figure 2:**
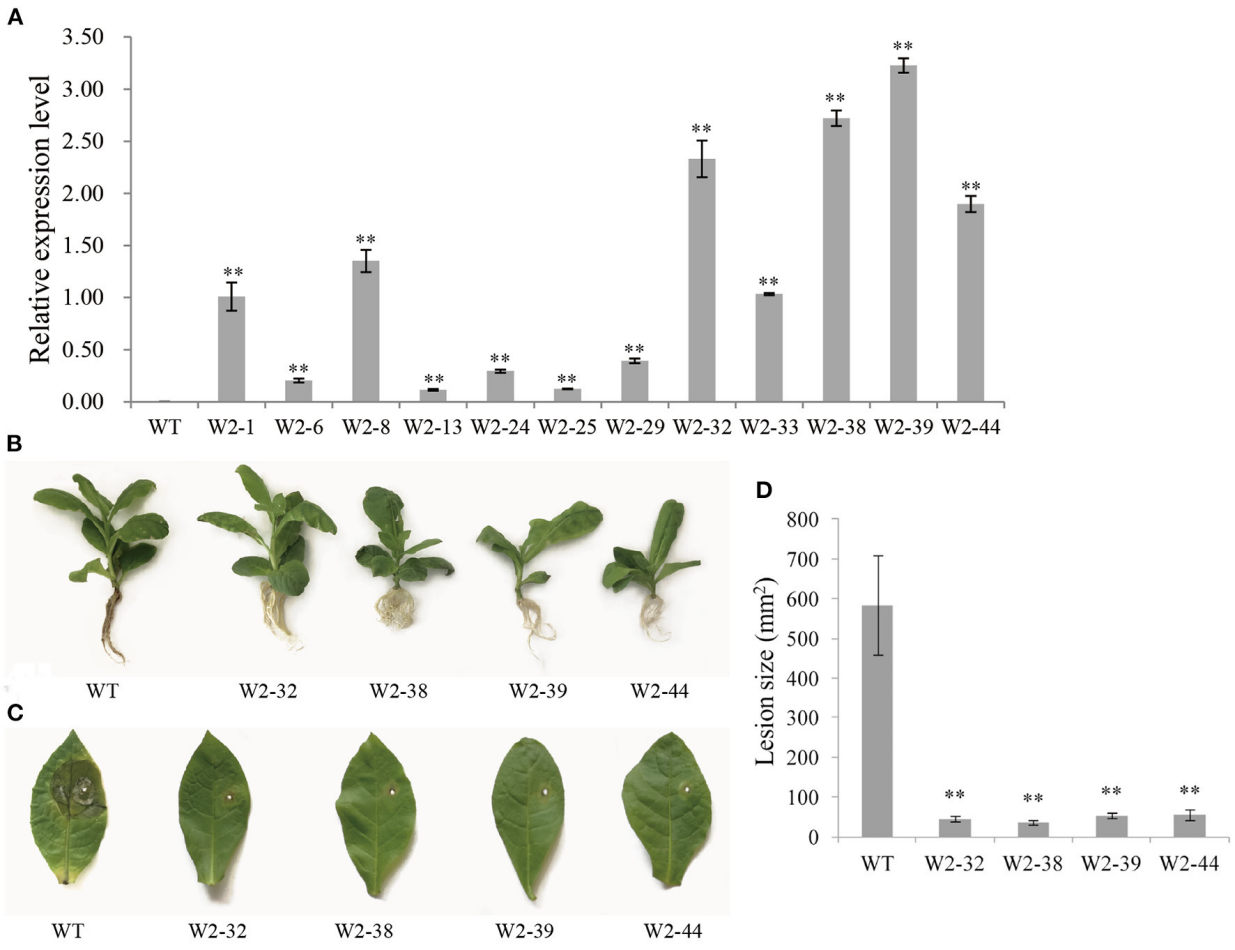
Gene expression and resistance analyses of T_2_ generation *LrWRKY2* transgenic tobacco lines. **(A)** The *LrWRKY2* was stably expressed in the 12 T_2_ generation *LrWRKY2* transgenic tobacco lines (W2-1/6/8/13/24/25/29/32/33/38/39/44). **(B)** The root inoculation assay revealed the enhanced resistance of four T_2_ *LrWRKY2* transgenic tobacco lines (W2-32/38/39/44) to *F. oxysporum* infection. **(C)** The leaf inoculation assay revealed the increased resistance of four T_2_ *LrWRKY2* transgenic tobacco lines (W2-32/38/39/44) to *F. oxysporum* infection. **(D)** The lesion areas in the inoculated leaves of the four T_2_ *LrWRKY2* transgenic tobacco lines were significantly smaller than that in the WT (**p < 0.01). Bars represent the standard errors of three biological replicates, and the statistical analysis was performed with the *t* test (**p < 0.01).

The integration of *LrWRKY2* into transgenic tobacco plants was verified by PCR. There were no differences in the phenotype and growth status of the WT and *LrWRKY2* transgenic tobacco plants. Twelve confirmed transgenic lines were selected for the subsequent investigation. The analysis of gene expression by qRT-PCR indicated that *LrWRKY2* was stably expressed in the transgenic lines (Figure 2A). Among the transgenic lines, *LrWRKY2* expression levels were highest in W2-32, W2-38, W2-39, and W2-44. Thus, these four lines were selected for further analyses.

The roots of WT and *LrWRKY2* transgenic tobacco plants were inoculated with an *F. oxysporum* spore suspension (5 × 10^6^ spores/mL). After a 7-day incubation, the inoculated WT plants had curled, yellow, and withered leaves as well as blackened or rotted roots. In contrast, the *LrWRKY2* transgenic tobacco leaves and roots were healthy and growing well (Figure 2B). The leaf inoculation assay produced similar results. More specifically, obvious disease symptoms were detected surrounding the inoculation site of the WT leaves (Figure 2C). The WT leaves were yellow and rotted, which was in sharp contrast to the healthy *LrWRKY2* transgenic leaves. The average leaf lesion size for the transgenic lines and WT control was <100 mm^2^ and ~580 mm^2^, respectively (Figure 2D). These results imply that *LrWRKY2* overexpression can substantially increase the resistance of transgenic tobacco to *F. oxysporum*.

**Figure 3:**
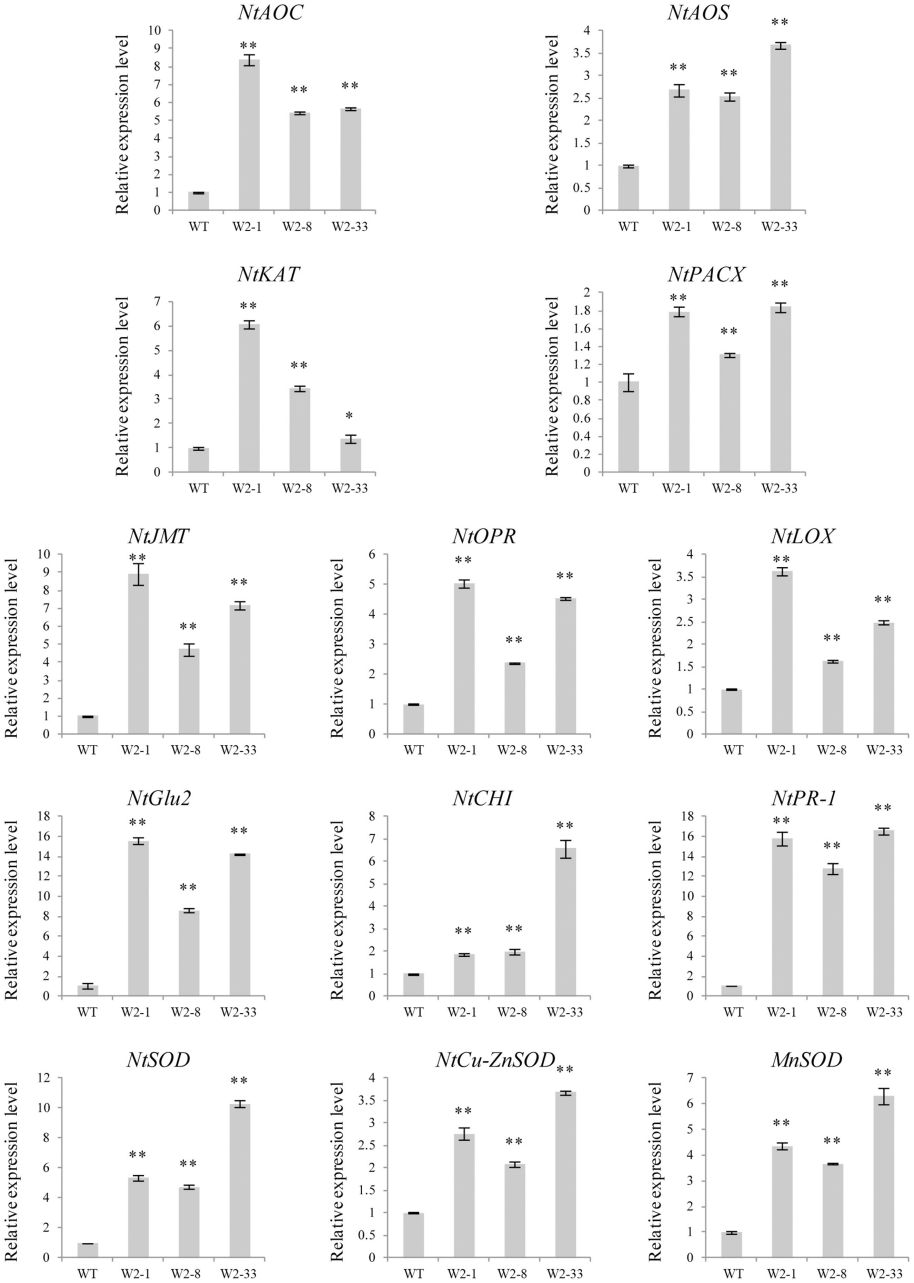
The expression levels of some JA biosynthesis and defense-related genes including the *NtAOC, NtAOS, NtKAT, NtPACX, NtJMT, NtOPR, NtLOX, NtCHI, NtGlu2, NtPR-1, NtSOD, NtCu-ZnSOD*, and *MnSOD* in the three T2 *LrWRKY2* transgenic tobacco lines (W2-1/8/33) were evaluated by qRT-PCR. Bars represent the standard errors of three biological replicates. The results was calculated by the 2^−ΔΔCt^ method and analyzed by the *t* test (*p < 0.05, **p < 0.01).

Several defense response-related genes were selected to examine the transcriptional changes in the *LrWRKY2* transgenic tobacco lines. The JA biosynthetic pathway-related genes (*NtAOC, NtAOS, NtKAT, NtPACX, NtJMT, NtOPR*, and *NtLOX*), *PR* genes (*NtCHI, NtGlu2*, and *NtPR-1*), and SOD genes (*NtSOD, NtCu-ZnSOD*, and *MnSOD*) showed higher expression levels in the *LrWRKY2* transgenic lines than in the WT plants (Figure 3). The JA biosynthetic pathway-related genes *NtAOC, NtKAT, NtJMT, NtOPR*, and *NtLOX* as well as the *PR* gene *NtGlu2* were most highly expressed in line W2-1, with the *NtGlu2* expression level 15-fold higher than that in the WT plants. The JA biosynthetic pathway-related genes *NtAOS* and *NtPACX*, the *PR* genes *NtCHI* and *NtPR-1*, and the SOD genes *NtSOD, NtCu-ZnSOD*, and *MnSOD* displayed the highest expression levels in line W2-33, with the *NtPR-1* expression level 17-fold higher than that in the WT plants. The expression levels of all analyzed genes were higher in line W2-8 than in the WT plants. These observations suggest that *LrWRKY2* overexpression in tobacco up-regulates the expression of JA signaling pathway genes and induces the expression of some PR and SOD genes.

### The Transient Expression of the Hairpin RNA Targeting *LrWRKY2* in *L. regale* Leads to Increased Susceptibility to *F. oxysporum*

**Figure 4:**
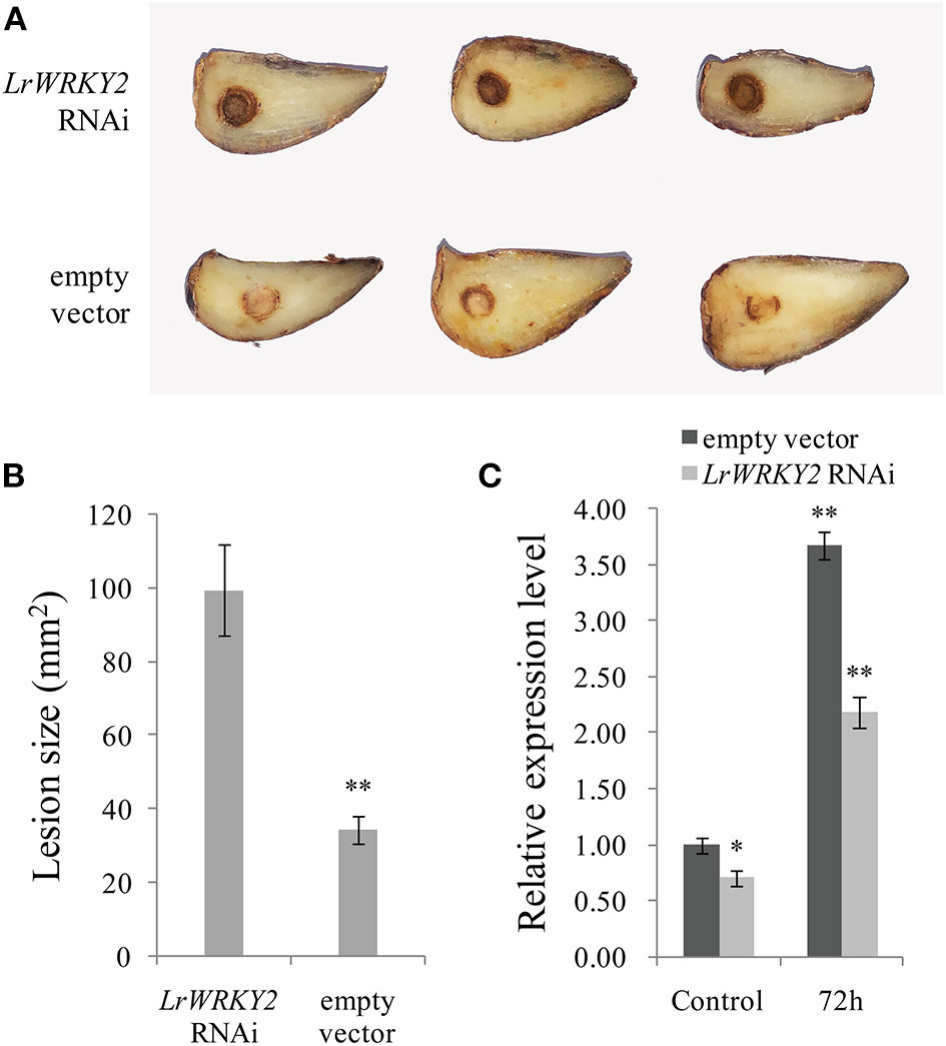
Analysis of the hairpin RNA targeting *LrWRKY2* transiently expressed in *L. regale* scales. **(A)** The symptoms of *L. regale* scales after *F. oxysporum* inoculation, in which the *LrWRKY2* RNAi vector and the empty RNAi vector were expressed, respectively. **(B)** The lesion areas in *LrWRKY2*-RNAi fragment expressed scales were significantly bigger than that in the empty RNAi vector expressed scales (**p < 0.01). **(C)** The expression levels of *LrWRKY2* in *LrWRKY2*-RNAi fragment expressed scales and the empty RNAi vector expressed scales were evaluated by qRT-PCR. Bars represent the standard errors of three biological replicates. The results was calculated by the 2^−ΔΔCt^ method and analyzed by the *t* test (*p < 0.05, **p < 0.01).

To clarify the effect of silencing *LrWRKY2* expression, the *LrWRKY2*-RNAi construct was transiently expressed in *L. regale* scales, which were then inoculated with *F. oxysporum*. After a 3-day incubation, the scales expressing the *LrWRKY2*-RNAi fragment had dark brown wounds and were rotted, whereas the scales transformed with the empty RNAi vector had light brown wounds and were only slightly rotted (Figure 4A). Moreover, the average lesion size was greater for the scales expressing the *LrWRKY2*-RNAi fragment (~100 mm^2^) than for the scales transformed with the empty RNAi vector (~28 mm^2^) (Figure 4B). At 3 days after the inoculation with *F. oxysporum*, the *LrWRKY2* expression level was significantly lower in the *L. regale* scales expressing the *LrWRKY2*-RNAi fragment than in the *L. regale* scales with the empty RNAi vector (Figure 4C). The lily scales expressing the *LrWRKY2*-RNAi fragment were more susceptible to *F. oxysporum* than the control scales, which was consistent with the relatively low *LrWRKY2* expression level due to the RNAi fragment. These findings imply that LrWRKY2 functions as a positive regulator of *L. regale* responses to an *F. oxysporum* infection.

### The *L. regale* Chitinase Gene *CHI2* Confers Resistance to *F. oxysporum*

To explore the regulatory effects of the LrWRKY2 TF on *PR* gene expression, an *L. regale* chitinase gene (*LrCHI2*) responsive to *F. oxysporum* was cloned on the basis of the *L. regale* transcriptome sequencing data (unpublished). Gene expression level of *LrCHI2* was estimated as Fragments Per Kilobase of transcript per Million mapped reads (FPKM). The FPKM of *LrCHI2* in *L. regale* roots without inoculation with *F. oxysporum* was 5.29, while its FPKM in *L. regale* roots inoculated with *F. oxysporum* for 96 h was 1015.59. Therefore, the log_2_FC (log_2_ fold changes) was 6.97, indicating a significant upregulation of *LrCHI2* gene in *L. regale* roots during *F. oxysporum* infection. The obtained full-length *LrCHI2* cDNA (accession no. MZ272344) consisted of 1,313-bp, with a 933-bp ORF, a 19-bp 5′ untranslated region, and a 361-bp 3′ untranslated region. The encoded protein comprising 310 amino acid residues was ~32.46 kDa and had an isoelectric point of about 5.65. A sequence analysis indicated the deduced LrCHI2 protein possesses a chitin-binding domain (ChtBD) and a Glyco_hydro_19 (GH19) catalytic domain. Additionally, a 491-bp *LrCHI2* promoter fragment (accession no. MZ272345) was obtained by TAIL-PCR. A number of *cis*-elements were identified in the *LrCHI2* promoter region, including ABRELATERD1 (abscisic acid-responsive element), GT1CONSENSUS (gibberellic acid-responsive element), and the W-box (Supplementary Table 3).

**Figure 5:**
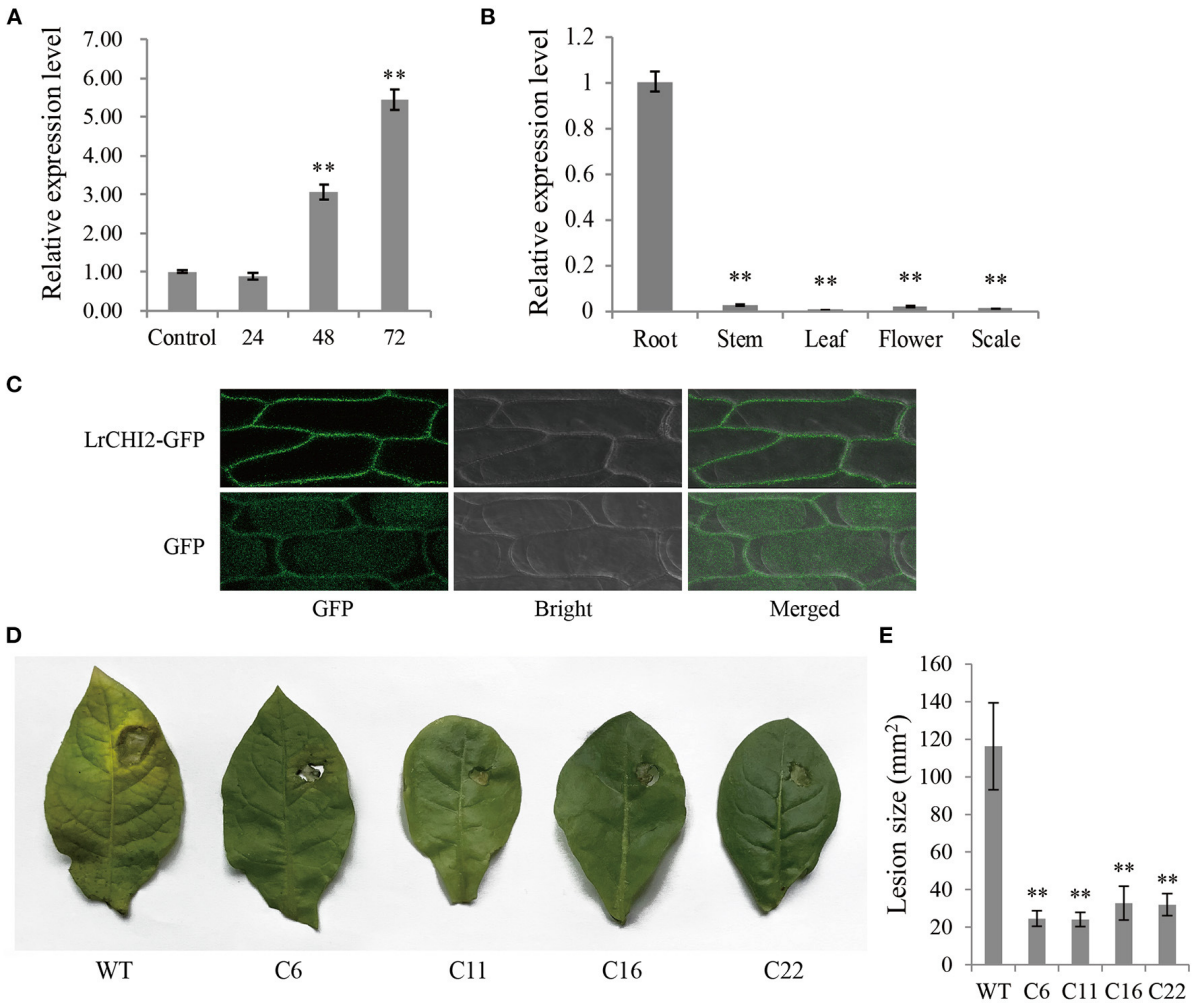
The functional analysis of *LrCHI2*. **(A)** The expression levels of *LrCHI2* in *L. regale* roots after inoculation with *F. oxysporum* were analyzed by qRT-PCR. The *L. regale* roots were inoculated with *F. oxysporum*, and then were collected at 4, 24, 48, and 72 hpi. The roots inoculated with sterile water were used as control sample. **(B)** The expression levels of *LrCHI2* in various tissues of *L. regale* under normal conditions were analyzed by qRT-PCR. **(C)** Subcellular localization analysis of LrCHI2-GFP fusion protein. The LrCHI2-GFP fusion protein was transiently expressed in cell wall after genetic transform mediated by *A. tumefaciens*. GFP, fluorescent light; Bright, white light; Merged, overlaid of fluorescent and white light. **(D)** The leaf inoculation assay revealed the four T_2_ *LrWRKY2* transgenic tobacco lines (C6, C11, C16, and C22) showed enhanced resistance to *F. oxysporum* infection. **(E)** The lesion areas in the inoculated leaves of the four T_2_ generation *LrCHI2* transgenic tobacco lines were significantly smaller than that in the WT (**p < 0.01). Bars represent the standard errors of three biological replicates. The statistical analysis was performed with the *t* test (**p < 0.01).

The qRT-PCR analysis indicated *LrCHI2* was expressed at relatively low levels in the stems, leaves, flowers, and scales (Figure 5A), but it was highly expressed in the roots. Moreover, the *F. oxysporum* infection significantly up-regulated *LrCHI2* expression in the *L. regale* roots (Figure 5B). Specifically, its expression level was about 3-fold and 5.5-fold higher than that in the control (not inoculated) at 24 and 48 h post-inoculation, respectively. Accordingly, *LrCHI2* appears to be predominantly expressed in the roots and its expression is induced by *F. oxysporum*.

An N-terminal signal peptide was detected in LrCHI2, indicating this protein is secreted. The subcellular localization of LrCHI2 was determined by expressing a GFP-tagged fusion protein in onion epidermal cells. The fluorescence of LrCHI2-GFP was exclusively detected in the cell wall, suggesting LrCHI2 is a cell wall protein (Figure 5C).

Four T_2_ transgenic tobacco lines overexpressing *LrCHI2* (C6, C11, C16, and C22) were randomly selected and examined regarding their resistance to *F. oxysporum*. The WT and *LrCHI2* transgenic tobacco leaves were inoculated with an *F. oxysporum* spore suspension (5 × 10^6^ spores/mL). After a 7-day incubation, transgenic tobacco leaves infected with *F. oxysporum* had relatively small wounds with slightly yellow edges, whereas the infected WT leaves had large wounds and were more obviously decayed (Figure 5D). Hence, *LrCHI2* overexpression considerably enhanced the resistance of the transgenic tobacco lines to *F. oxysporum*. The lesions on the leaves of the four transgenic tobacco lines (C6, C11, C16, and C22) were smaller than 40 mm^2^, whereas the WT leaf lesions were almost 120 mm^2^ (Figure 5E).

**Figure 6:**
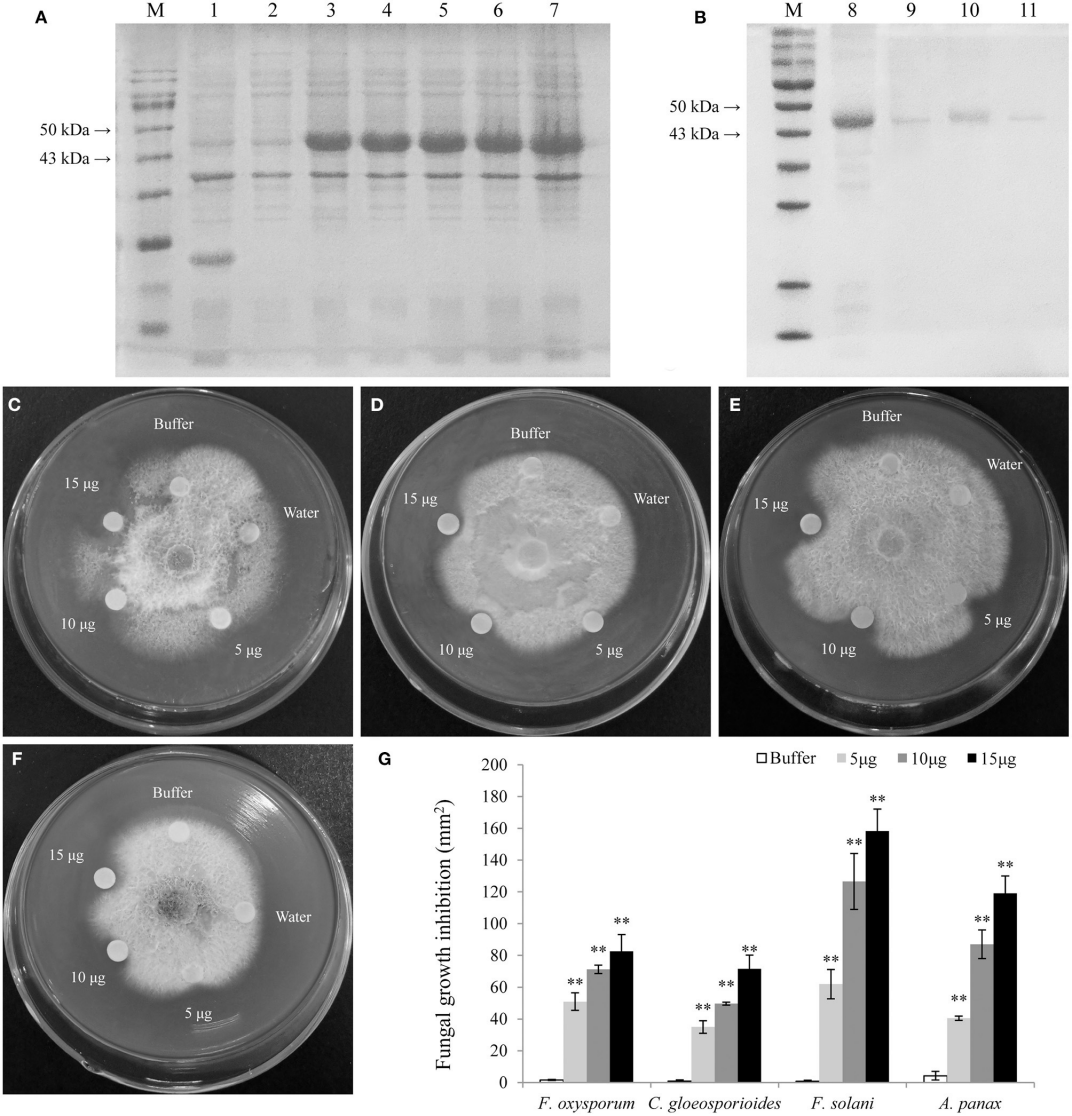
Expression, purification, and antifungal assay of LrCHI2 recombinant protein. **(A)** The induced expression of LrCHI2 recombinant protein under 1 mM IPTG condition. M, protein marker; Line 1, the protein of *E. coli* with empty vector pET-32a was detected after induction; Line 2, the expression of LrCHI2 protein was detected without induction; Line 3-7, the expression of LrCHI2 protein was detected at 6, 8, 10, 12, and 24 h after induction, respectively. **(B)** The purification of LrCHI2 recombinant protein. M, protein marker; Line 8, the supernatant after treatment with the inclusion body solubilizing solution; Line 9-11, the purified LrCHI2 recombinant protein with 50, 100, 150 mM imidazole washing buffer, respectively. **(C–F)** The LrCHI2 recombinant protein has evident antifungal activity to *F. oxysporum* **(C)**, *C. gloeosporioides* **(D)**, *F. solani* **(E)** and *A. panax* **(F)**. **(G)** The fungal growth inhibition areas (mm^2^). Bars represent the standard errors of three biological replicates. The statistical analysis was performed with the *t* test (**p < 0.01).

The LrCHI2 recombinant protein lacking a signal peptide was expressed in *E. coli* cells. The SDS-PAGE analysis indicated that the His-tagged LrCHI2 protein produced in cells induced by isopropyl-β-D-1-thiogalactopyranoside (IPTG) was ~47 kDa, which was consistent with the predicted size (Figure 6A). The LrCHI2 recombinant protein in inclusion bodies was solubilized and then purified by nickel-affinity chromatography, with most of the protein eluted by the 100 mM imidazole elution buffer. The lack of extra bands during the SDS-PAGE analysis reflected the purity of the obtained LrCHI2 recombinant protein (Figure 6B). The *in vitro* antifungal activity of the purified LrCHI2 recombinant protein was examined. Compared with the blank controls, the LrCHI2 recombinant protein significantly inhibited the growth of *F. oxysporum, C. gloeosporioides, F. solani*, and *A. panax* (Figures 6C–F). Moreover, the inhibitory effect increased as the concentration of the recombinant protein solution used to treat the tested fungi increased (Figure 6G).

### LrWRKY2 Binds to the *LrCHI2* Promoter Fragment Containing the W-box and Activates Transcription

**Figure 7:**
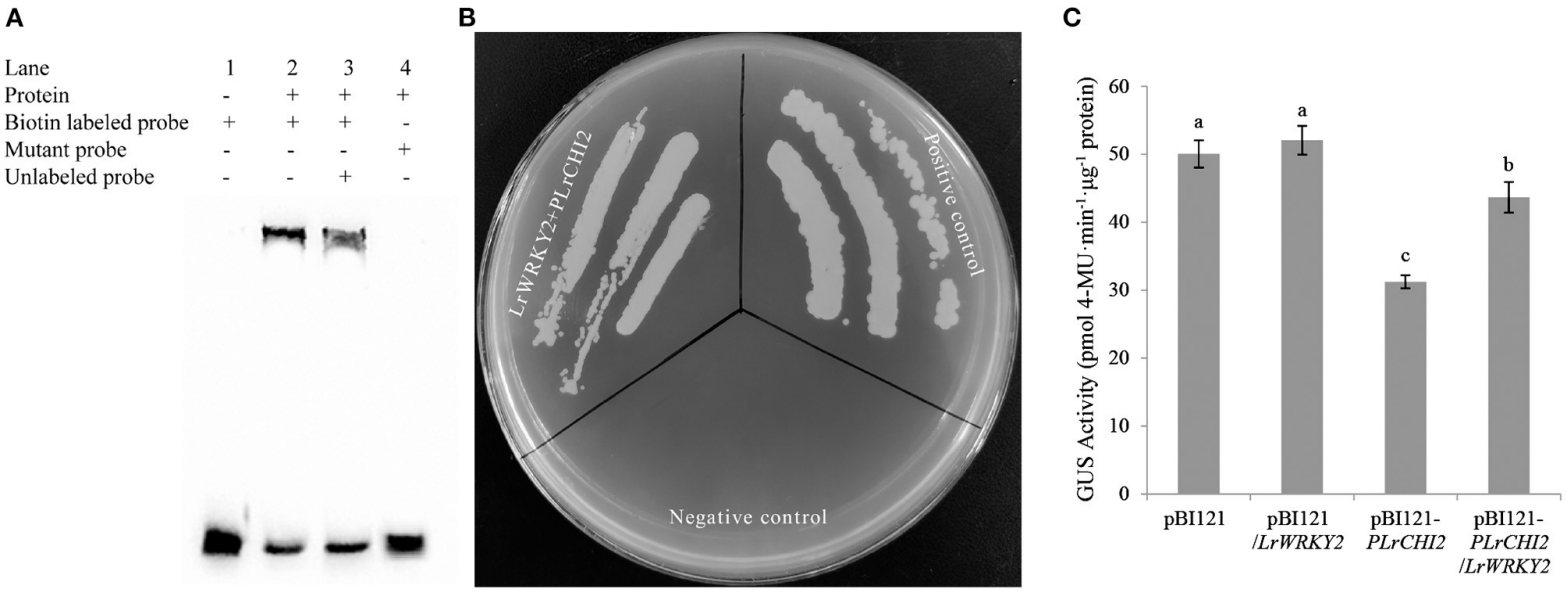
Analysis of the interaction between LrWRKY2 and *LrCHI2* promoter. **(A)** The specific binding of LrWRKY2 with the *LrCHI2* promoter fragment was verified by EMSA. Line 1, the biotin labeled probes containing the W-box sequence. Line 2, LrWRKY2 can interact with the biotin labeled probes containing the W-box sequence 3, LrWRKY2 can interact with both the biotin labeled and unlabeled probes containing the W-box sequence. 4, LrWRKY1 can't interact with mutant probes. **(B)** Analysis of the transactivation effect of LrWRKY2 recombinant protein on *LrCHI2* promoter. LrWRKY2 + pLrCHI2, the interaction of pGADT7-*LrWRKY2* and *LrCHI2* promoter; Positive control, the interaction of pGADT7-*p53* and pAbAi-*p53*; Negative control, the interaction of pAbAi-*p53* and pGADT7*-LrWRKY2*. **(C)** GUS activity was higher in transgenic tobacco lines co-expressing of *LrWRKY2* and *LrCHI2* promoter than in the transgenic tobacco lines expressing of *LrCHI2* promoter. Bars represent the standard errors of three biological replicates. The statistical differences were analyzed by Duncan's multiple range test (Different letters indicate a significant difference).

The WRKY TFs often target the W-box *cis*-element to activate or suppress target gene expression. The EMSA results confirmed the LrWRKY2 recombinant protein purified from *E. coli* cells was able to bind directly to the W-box sequence in the *LrCHI2* promoter. The biotin-labeled probes alone revealed a lack of band shifts in the gel (lane 1, Figure 7A). However, LrWRKY2 was able to bind to the biotin-labeled probes at the *LrCHI2* promoter fragment, resulting in a mobility shift (lane 2, Figure 7A). The inclusion of both biotin-labeled and unlabeled probes resulted in the detection of a band shift in the gel (lane 3, Figure 7A), reflecting the binding of the two probes to LrWRKY2. Because the unlabeled probes were 50-fold more abundant than the biotin-labeled probes, LrWRKY2 mainly bound to the competitor (unlabeled probe). Thus, the band shift was less intense in lane 3 than in lane 2 (Figure 7A). A band shift was undetectable in lane 4 (Figure 7A), which corresponded to the analysis involving the mutant probe and LrWRKY2, implying LrWRKY2 binds specifically to the W-box in the *LrCHI2* promoter.

The ability of LrWRKY2 to activate transcription was evaluated in a Y1H assay, in which Y1HGold yeast cells were transformed with pGADT7-*LrWRKY2* and *pLrCHI2*-pAbAi. The assay results indicated that the yeast cells co-transformed with pGADT7-*LrWRKY2* and *pLrCHI2*-pAbAi grew well, whereas the yeast cells transformed with pGADT7-*LrWRKY2* and the empty pAbAi vector failed to survive on the SD/–Leu selective medium containing 50 ng/mL AbA (Figure 7B). These observations indicate LrWRKY2 can bind to the *LrCHI2* promoter *in vivo* and activate transcription in yeast cells.

The transcriptional activation of *LrCHI2* by LrWRKY2 was verified by inserting an expression construct comprising a GUS-encoding gene under the control of the *LrCHI2* promoter into *LrWRKY2* transgenic tobacco. A total of 23 transgenic tobacco plants carrying both *LrWRKY2* and *pLrCHI2-GUS* and 27 transgenic tobacco plants transformed with *pLrCHI2-GUS* alone were identified by PCR. The GUS activity in the *pLrCHI2-GUS* transgenic tobacco plants was significantly lower than that in the transgenic tobacco carrying both *LrWRKY2* and *pLrCHI2-GUS* (Figure 7C). The activity of the GUS expressed under the control of the *LrCHI2* promoter increased by approximately 1.4-fold in the presence of LrWRKY2. There were no significant differences in the GUS activities of the two kinds of transgenic tobacco plants transformed with the empty pBI121-*GUS* vector. This implies that LrWRKY2 does not affect the CaMV 35S promoter. Therefore, LrWRKY2 positively regulates *LrCHI2* expression.

## Discussion

The WRKY proteins are critical plant TFs mediating diverse biological processes. However, little is known about the functions of *L. regale* WRKY genes. Protein functions are closely related to the distribution of the proteins in cells. The localization of LrWRKY2 in the nucleus is in accordance with its putative function as a TF that must exist in the nucleus to regulate the transcription of target genes (Liu et al., 2013; Wang et al., 2013; Sun et al., 2015). Similarly, maize (*Zea mays*) WRKY106 (Wang C. T. et al., 2018) and cotton (*Gossypium hirsutum*) WRKY33 are also nuclear proteins (Wang et al., 2019). In fact, the vast majority of the WRKY TFs identified to date are localized in the nucleus. Accordingly, LrWRKY2 is a nuclear protein that regulates cellular processes. In contrast, LrCHI2 was localized to the cell wall in this study. The plant cell wall is the first line of defense against a pathogen invasion. It is possible that LrCHI2 participates in chitinase–pathogen interactions. Some *Cucumis sativus* chitinases are also localized in the cell wall, wherein they interact directly or indirectly with pathogen elicitors to trigger downstream defense pathways (Bartholomew et al., 2019).

The LrWRKY2 TF belongs to Subgroup IIe of the WRKY family (Li et al., 2021a). Previous research proved that many Subgroup IIe WRKY TFs positively regulate plant responses to biotic and abiotic stresses. For example, the overexpression of the Subgroup IIe *CmWRKY10* chrysanthemum (*Chrysanthemum morifolium*) gene reportedly leads to increased drought tolerance (Jaffar et al., 2016). Transgenic tobacco plants overexpressing the *C. annuum WRKY27* gene enhances the resistance to *Ralstonia solanacearum* (i.e., milder disease symptoms and inhibited pathogen growth) (Dang et al., 2014). Herbaceous peony (*Paeonia lactiflora*) plants in which the Subgroup IIe gene *WRKY65* is silenced exhibit increased susceptibility to *Alternaria tenuissima*, suggesting PlWRKY65 is a positive regulator of peony defense responses to *A. tenuissima* (Wang et al., 2020). It is well-known that the lily is difficult to perform the genetic transform, and only a few lily transformations have been achieved thus far (Yan et al., 2019b). The currently established lily transformation system still has some problems, such as the strong genotype dependence, low efficiency of stable transformation, poor genetic stability, and difficult regeneration after transformation (Yan et al., 2019b; Song et al., 2020). It is not so bad that the *Agrobacterium*-mediated transformation of tobacco using leaf disks has provided a valuable tool for rapid evaluation of function of the transgenes in higher plants (Clemente, 2006). Therefore, the stable genetic transformation of *LrWRKY2* was completed in the model plant tobacco in order to further understand the function of LrWRKY2 in the present study. The *LrWRKY2*-overexpressing transgenic tobacco plants were more resistant to *F. oxysporum* than the WT control plants. Additionally, *L. regale* scales transiently expressing *LrWRKY2*-RNAi were more susceptible to *F. oxysporum* than the control scales. Our results suggest that LrWRKY2 is an important TF influencing *L. regale* disease resistance.

The ectopic expression of TF genes facilitated by transgenic technology and the subsequent analysis of the transcriptional changes in the generated transgenic plants may provide new insights regarding transcriptional regulatory networks. As crucial components of plant defense systems, WRKY proteins regulate the expression of some defense-related genes. The overexpression of rice Subgroup IIa WRKY genes (*OsWRKY62, OsWRKY28, OsWRKY71*, and *OsWRKY76*) up-regulates the *PR10* expression level (Peng et al., 2010). The constitutive overexpression of *PtrWRKY18* and *PtrWRKY35* in poplar (*Populus trichocarpa*) enhances the resistance to the biotrophic pathogen *Melampsora* by inducing *PR* gene expression (Jiang et al., 2017). The ectopic overexpression of the cotton (*G. hirsutum*) *WRKY44* gene increases the resistance of *Nicotiana benthamiana* plants to fungal infections and activates the expression of *PR* genes, including *PR1a, PR4, PR5*, and *NPR1* (Li et al., 2015). The ectopic expression of rice *OsWRKY11* results in the constitutive expression of defense-associated genes (*CHIT2, PR10*, and *Betv1*), whereas down-regulating *OsWRKY11* expression adversely affects the expression of defense-related genes during pathogen invasions, suggesting that OsWRKY11 activates defense responses (Lee et al., 2018). The heterologous expression of rice *OsWRKY6* in *A. thaliana* can dramatically induce the expression of defense-related genes, including *PR1, PDF1, NPR4*, and a glucanase gene (Hwang et al., 2011). In the present study, the expression levels of several *PR* genes (e.g., *NtCHI, NtGlu2*, and *NtPR-1*) were clearly up-regulated in *LrWRKY2* transgenic tobacco lines. Additionally, the expression levels of JA biosynthetic pathway-related genes (*NtAOC, NtAOS, NtKAT, NtPACX, NtJMT, NtOPR*, and *NtLOX*) were higher in the *LrWRKY2*-overexpressing transgenic tobacco lines than in the WT tobacco plants, implying that the JA signaling pathway was activated in response to *LrWRKY2* overexpression. Consistent with our findings, a previous study revealed that in transgenic *A. thaliana* lines overexpressing an *L. regale* WRKY gene, the expression levels of some JA-responsive genes, including *AtLOX, AtMYC2*, and *AtPDF1.2*, are up-regulated (Cui et al., 2018). Moreover, overexpressing *PtrWRKY40* in *A. thaliana* activates the expression of JA-related defense genes, ultimately leading to the resistance to the necrotrophic fungus *Botrytis cinerea* (Karim et al., 2015). Furthermore, the expression levels of SOD genes (*NtSOD, NtCu-ZnSOD*, and *MnSOD*) increased significantly in the *LrWRKY2*-overexpressing tobacco lines, suggesting that LrWRKY2 confers enhanced disease resistance by activating the JA signaling pathway and inducing the expression of defense-related genes.

The PR proteins have important functions related to plant defense responses to pathogens. They accumulate after pathogen invasions, and may act as antimicrobial agents mediating cell wall hydrolysis, contact toxicity, and perhaps defense signaling (Zhang et al., 2017). A recent study demonstrated that *Panax notoginseng* PR-like proteins can inhibit *F. solani* and *C. gloeosporioides* mycelial growth (Li et al., 2021b). Another study proved that overexpressing the moss (*Physcomitrella patens*) *PR10* gene in *A. thaliana* enhances plant resistance to *Pythium irregulare* (Castro et al., 2016). In the current study, *LrCHI2* isolated from *L. regale* was characterized as a new *PR* gene. This gene was mainly expressed in the roots, and its expression was induced by an *F. oxysporum* infection. Additionally, LrCHI2 exhibited *in vitro* antifungal activity that inhibited *F. oxysporum, C. gloeosporioides, F. solani*, and *A. panax* mycelial growth. The overexpression of *LrCHI2* in tobacco enhanced the resistance of the transgenic plants to *F. oxysporum*. Thus, *LrCHI2* expression contributes to the resistance of *L. regale* to *F. oxysporum*.

Previous research proved that various *PR* genes are targeted by WRKY TFs, which bind to the canonical W-box sequence (TTGACC/T) in the gene promoters. A recent Y1H assay confirmed that rice OsWRKY6 can bind directly to the W-box derived from the rice *PR1* promoter in yeasts (Hwang et al., 2011). An *in vivo* chromatin immunoprecipitation assay and *in vitro* EMSA experiments revealed that *C. annuum* WRKY40 binds directly to a *C. annuum* defensin gene promoter and the *WRKY33* promoter, both of which contain a W-box; on the contrary, with mutations to the W-box sequence preventing the binding (Chakraborty et al., 2019). The LrWRKY1 TF induces the transcription of *LrPR10-5*, which has a promoter containing three W-boxs (Li et al., 2021a). The co-expression of *LrWRKY1* and *LrPR10-5* in tobacco indicated LrWRKY1 can activate the *LrPR10-5* promoter. In this study, the regulatory effect of LrWRKY2 on the *LrCHI2* promoter was examined. This promoter contains hormone-responsive, biotic and abiotic stress-related elements as well as one W-box. The EMSA results proved that LrWRKY2 has a high *in vitro* affinity for the *LrCHI2* promoter containing a W-box. Additionally, the Y1H assay results verified the interaction between LrWRKY2 and the *LrCHI2* promoter fragment in yeast. These findings suggest that LrWRKY2 binds to the W-box in the *LrCHI2* promoter to activate expression. This likely contributes to the LrWRKY2-regulated *L. regale* defense response to *F. oxysporum*. Because the transcript levels of several *PR* genes, including *NtCHI, NtGlu2*, and *NtPR-1*, increased in *LrWRKY2* transgenic tobacco lines, future studies should investigate whether LrWRKY2 regulates the expression of other *PR* genes in *L. regale*.

## Conclusions

The nuclear protein LrWRKY2 belongs to Subgroup IIe of the WRKY family. Transgenic tobacco lines overexpressing *LrWRKY2* are highly resistant to *F. oxysporum*, which may be related to the significantly up-regulated expression of many defense-related genes, including JA biosynthetic pathway-related genes, *PR* genes, and SOD genes. Additionally, the transient expression of the *LrWRKY2*-RNAi fragment in *L. regale* scales leads to increased sensitivity to *F. oxysporum*. Moreover, LrWRKY2 is a positive regulator that mediates *L. regale* defense responses to *F. oxysporum* infection by regulating the chitinase gene expression, which is a broad-spectrum resistance gene showing antifungal activity to some important phytopathogens including *F. oxysporum*. The results of this study may be relevant for future research aimed at elucidating the transcriptional regulatory mechanisms underlying the interaction between *L. regale* and *F. oxysporum*. In addition, the lily genetic transformation mediated by the *Agrobacterium* has been carried out in our research group. It is believed that a more in-depth understanding of the defense response regulation mechanism in *L. regale* against the Fusarium wilt will be obtained in the near future.

## Data Availability Statement

The datasets presented in this study can be found in online repositories. The names of the repository/repositories and accession number(s) can be found in the article/Supplementary Material.

## Author Contributions

SL: methodology, data curation, experiment execution, and writing—original draft preparation. JH: methodology, material collection, and supervision. ZW: investigation and software. JD: validation. TL: supervision. LS: conceptualization. DL: methodology, supervision, writing—reviewing, and editing. All authors contributed to the article and approved the submitted version.

## Funding

This work was financially supported by two grants received from the National Natural Sciences Foundation of China (grant number 31760586) and Yunnan Ten Thousand Talents Plan Young & Elite Talents Project, respectively.

## Conflict of Interest

The authors declare that the research was conducted in the absence of any commercial or financial relationships that could be construed as a potential conflict of interest.

## Publisher's Note

All claims expressed in this article are solely those of the authors and do not necessarily represent those of their affiliated organizations, or those of the publisher, the editors and the reviewers. Any product that may be evaluated in this article, or claim that may be made by its manufacturer, is not guaranteed or endorsed by the publisher.
