# Supplementary material for: Lilium regale Wilson WRKY2 Regulates Chitinase Gene Expression During the Response to the Root Rot Pathogen Fusarium oxysporum
Source: Front Plant Sci. 2021 Sep 27;12:741463. doi: 10.3389/fpls.2021.741463 (PMC8503523; doi:10.3389/fpls.2021.741463)
Supplement: Supplementary file 1 [file Table_1.docx]

**Supplementary Tables:**

Table S1 The primers’ sequences used for functional analyses of *LrWRKY2* and *LrCHI2*

| Target genes | Primer sequences | Experiments |
| --- | --- | --- |
| *LrCHI2* | Forward: 5’ ATGGCAGCACCAAAGCTTCT 3’  Reverse: 5’ GAAGTGCTGCTGGTTGTAGCAG 3’ | Gene cloning |
| *LrCHI2* | Forward: 5’ CCATATGGATGGCAGCACCAAAGCTTCTTC 3’  Reverse: 5’ CGGGATCCCGCTGCTACAACCAGCAGCACTTC 3’ | Subcellular localization |
| *LrCHI2* | Forward: 5’ GATATCATGCAGCAATGCGGCAGCCAAGG 3’  Reverse: 5’ GGAATTCCTCAGAAGTGCTGCTGGTTGTAGCAGT 3’ | Prokaryotic expression |
| *LrCHI2* | Forward: 5’ GGAATTCCATGGCAGCACCAAAGCTTCTTC 3’  Reverse: 5’ CGGGATCCCGTCAGAAGTGCTGCTGGTTGTAGC 3’ | Overexpression |
| *pLrCHI2* | Reverse1: 5’ CATCATAGGTGTAGAAGCCGTTCGCAG 3’  Reverse2: 5’ GGCTGCCGCATTGCTGAGCAG 3’ | Genome-walking |
| *pLrCHI2* | Forward: 5’ CAAGCTTGTCATACGTGTGTCCCCTATCATG 3’  Reverse: 5’ CCCGGGGCTTTGGTGCTGCCATTGTAT 3’ | Yeast one-hybrid |
| *pLrCHI2* | Forward: 5’ CCCAAGCTTGGGTCATACGTGTGTCCCCTATCATG 3’  Reverse: 5’ CGGGATCCCGGCTTTGGTGCTGCCATTGTAT 3’ | Promoter cloning |
| *LrWRKY2* | Forward: 5’ CATATGTGTGATCTCTTCTGGCAAA 3’  Reverse: 5’ CCCGGGCGGCCCATTCAAACA 3’ | Subcellular localization |
| *LrWRKY2* | Forward: 5’ CCCAAGCTTGGGCTCCCTCTCTCACCCCTACA 3’  Reverse: 5’ CGCGGATCCGCGTCTATGACTCCAGATTATTT 3’ | Prokaryotic expression |
| *LrWRKY2* | Forward: 5’ TCCCCCGGGGGAATGTGTGATCTCTTCTGGCA 3’  Reverse: 5’ CCGCTCGAGCGGTTATTTCCCGGCCCATT 3’ | Yeast one-hybrid |
| *LrWRKY2* | Forward: 5’ CGGGATCCCGCTACAAGGTTCATCCTGCTAGACAT 3’  Reverse: 5’ GCTCTAGAGCTTTGCTTTCCATCCACTAGATAACA 3’ | Overexpression |
| *LrWRKY2* | Forward: 5’ GGGGACAAGTTTGTACAAAAAAGCAGGCTCTTTGGGCTTGGAGGAAGTATGG 3’  Reverse: 5’ GGGGACCACTTTGTACAAGAAAGCTGGGTCGAGTCTGGTATCATTGGCTTGTA 3’ | RNAi |
| Probe | GGCAAACTTTTAGGCCAAGAGATTGACTAGAACCCTCACAATCTCTATAT | EMSA |
| Mutant probe | GGCAAACTTTTAGGCCAAGAGATTACCTAGAACCCTCACAATCTCTATAT | EMSA |
| GUS | Forward: 5’ CGGATACCCGTCCGCAAGT 3’  Reverse: 5’ GTGTGAGCGTCGCAGAACATT 3’ | Co-expression |

Table S2 The primers’ sequences used for qRT-PCR

| Target genes | Primer sequences |
| --- | --- |
| *LrWRKY2* | Forward: 5’ GATCTTTGGGCTTGGAGGAAGT 3’  Reverse: 5’ TGTCGATCCAGCGAGAGCGT 3’ |
| *LrCHI2* | Forward: 5’ CAACTACGGCCCAGCTGGAC 3’  Reverse: 5’ TGTCGGCACCATGTCCACAC 3’ |
| *NtAOC*  (AJ308487.1) | Forward: 5’ AAGAAGAGAATTGGAATAACGGCT 3’  Reverse: 5’ GATCCCTGAACGGCGATGT 3’ |
| *NtAOS*  (AB778304.1) | Forward: 5’ CCACCAGTTGCTTCTCAATACGG 3’  Reverse: 5’ GAACTCATCGGGTCGGTCAAA 3’ |
| *NtKAT*  (XM_016651715.1) | Forward: 5’ TTGTTGATCCAAAAACCGGAGA 3’  Reverse: 5’ TGGCTAGAGTTCCCAGCAGTAGTAG 3’ |
| *NtPACX* (KJ730264.1) | Forward: 5’ AAGCAGCTAAGTTAAGGCATTTTGTA 3’  Reverse: 5’ GTTCAGTTTGAGCGTAGCACCCA 3’ |
| *NtJMT* (XM_016639122.1) | Forward: 5’ TTGGGTACTGAAGCAAGGACAGC 3’  Reverse: 5’ GCTCCTCCCCATTAACGACAAC 3’ |
| *NtOPR* (XM_016592560.1) | Forward: 5’ CGTTCATTTGTAGTGGCGGAT 3’  Reverse: 5’ CCTCATAACCAAATCAGGATTAGAAA 3’ |
| *NtLOX*  (X84040.1) | Forward: 5’ TGGTTATCTCCCTAATCGCCCTAC 3’  Reverse: 5’ CGAGGTAAAGTGTATCCGAAGAATG 3’ |
| *NtGlu2*  (A16120.1) | Forward: 5’ TTGATGCCCTTTTGGATTCTATG 3’  Reverse: 5’ TTTCCAGGTTTCTTTGGAGTTCC 3’ |
| *NtPR-1*  (X05454.1) | Forward: 5’ AGAACCTTTGACCTGGGACGAC 3’  Reverse: 5’ ATCCAACACGAACCGAGTTACG 3’ |
| *NtCHI*  (A16119.1) | Forward: 5’ ACGGACCTTGTGGAAGAGCCAT 3’  Reverse: 5’ ACCAAATCCAGGGAGACGATTG 3’ |
| *NtSOD*  (EU342358.1) | Forward: 5’ TGAAGCTGGTGGTCAATACATGG 3’  Reverse: 5’ GAGACATTGTCTTATATTGGAAGAGGA 3’ |
| *NtCu-ZnSOD* (EU123521.1) | Forward: 5’ CATGGTGCTCCTGAAGATGAGGT 3’  Reverse: 5’ CAGCATTTCCAGTAGCTTTACTGAG 3’ |
| *MnSOD*  (X14482.1) | Forward: 5’ GTGTGGCTTGGTGTGGACAAAG 3’  Reverse: 5’ CCTCAAAACAACATCAAATATCCCTG 3’ |
| *NtACT*  (AB158612.1) | Forward: 5’ TCCCATTGAGCATGGAATAGTAAGC 3’  Reverse: 5’ TACATGGCAGGTACATTGAAAGTCT 3’ |
| *LrGAPDH*  (KJ543468.1) | Forward: 5’ ACTTGGTTTCCACTGATTTCCTCG 3’  Reverse: 5’ CTTGCTAATGTGGCGGATGAGAT 3’ |

Table S3 The predicted cis-acting elements in the promoter sequence of *LrCHI2*

| The name of *cis*-element | sequence | Elements function | Position (bp) |
| --- | --- | --- | --- |
| WBOXNTERF3 | TGACT | Wound stress response element | -49~-53 |
| W-box | TTGACT | ET、SA、MeJA response element | -49~-54 |
| WBOXATNPR1 | TTGAC | SA response element | -50~-54 |
| WRKY71OS | TGAC | GA_3_ response element | -51~54 |
| ABRELATERD1 | ACGTG | ABA response element；high salt and dark inducing element | -89~--93  -482~-486 |
| SEBFCONSSTPR10A | TTGTCTC | Auxin-like response element | -106~-112 |
| EBOXBNNAPA | CAGTTG | ABA response element | -110~-115  -399~-404 |
| ROOTMOTIFTAPOX1 | ATATT | Root-specific regulatory element | -182~-186  -250~-254 |
| MYBCORE | CTGTTA | Water stress response element | -191~-196 |
| SORLIP1AT | GCCAC | Light control element | -228~-232  -328~-332 |
| SURECOREATSULTR11 | GAGAC | Auxin response factor binding site | -379~-383 |
| IBOXCORE | GATAA | Light control element | -458~-462 |
